# Nomogram for predicting risk of arm lymphedema following axillary lymph node dissection in breast cancer patients

**DOI:** 10.3389/fonc.2025.1667939

**Published:** 2025-11-21

**Authors:** Miaomiao Jia, Lihui Pan, Haibo Yang, Jinnan Gao, Wenzhuang Shen, Xiaojun Zhang

**Affiliations:** 1 Department of Breast Surgery, Shanxi Bethune Hospital, Shanxi Academy of Medical Sciences, Third Hospital of Shanxi Medical University, Tongji Shanxi Hospital, Taiyuan, China; 2 Tongji Hospital, Tongji Medical College, Huazhong University of Science and Technology, Wuhan, China

**Keywords:** breast cancer, lymphedema, risk factors, neoadjuvant chemotherapy, nomogram

## Abstract

**Purpose:**

Breast cancer-related arm lymphedema (BCRaL) is a prevalent and severe complication post-breast cancer treatment, especially following axillary lymph node dissection (ALND). This study aimed to develop a nomogram for BCRaL risk prediction by identifying and integrating key risk factors, including chemotherapy type (neoadjuvant vs. adjuvant), to enhance individualized patient monitoring and prevention strategies.

**Patients and methods:**

We conducted a retrospective analysis of clinical data from 535 breast cancer patients who received ALND and chemotherapy. Patients were divided into a training cohort (70%) and a validation cohort (30%). Univariate and multivariate Cox regression analyses identified independent risk factors for BCRaL, which were subsequently used to construct a nomogram. The model’s performance was assessed through calibration curves, ROC curves, and clinical decision curve analysis (DCA).

**Results:**

The incidence of BCRaL in our cohort was 20.6%. Multivariate analysis identified several independent risk factors for BCRaL, including elevated body mass index (BMI), increased number of positive axillary lymph nodes, neoadjuvant chemotherapy (NAC), HER2-targeted therapy, and supraclavicular radiotherapy (SCRT). The nomogram developed based on these factors demonstrated strong predictive accuracy, with C-index values of 0.692 in the training cohort and 0.719 in the validation cohort. ROC curve analysis revealed AUC values reaching 0.760, indicating good discriminative ability. Time-dependent ROC curves further confirmed the model’s consistency across different follow-up periods. DCA validated the clinical utility of the nomogram, while survival analysis clearly distinguished between high-risk and low-risk BCRaL groups.

**Conclusion:**

This study developed and internally validated a predictive model that integrates modern treatment modalities (NAC, HER2-targeted therapy, SCRT) with traditional risk factors to identify high-risk BCRaL patients undergoing ALND and chemotherapy. The model requires external validation in future studies. Consequently, the nomogram presents a potential tool for strategizing precision prevention, necessitating further evaluation before its broader adoption in clinical practice.

## Introduction

Breast cancer-related arm lymphedema (BCRaL) is a prevalent and serious complication following breast cancer treatment. Axillary lymph node dissection (ALND), a key surgical procedure for breast cancer, is the primary cause of BCRaL, affecting 19.9% of patients, compared to 5.6% following sentinel lymph node biopsy (SLNB) (1). BCRaL induces upper limb heaviness, pain, numbness, and dysfunction, along with psychological issues like anxiety, depression, and suicidal tendencies, significantly impacting patients’ quality of life (2–6). Although BCRaL cannot be cured, existing interventions can alleviate the symptoms of early-stage BCRaL patients but are ineffective in treating patients with late-stage BCRaL (7, 8). Effective prediction and early identification of high-risk BCRaL patients are crucial for prevention and early intervention.

Several risk prediction models have been proposed to assess BCRaL risk, and the risk factors identified vary slightly between models, depending on the study population, sample size, and variables included in the analysis (9–13). Risk factors for BCRaL that are supported by the evidence include high body mass index (BMI), removal of increased lymph nodes, and receiving modified radical mastectomy (MRM), ALND, radiotherapy, or chemotherapy (14–19). Breast cancer treatment is becoming increasingly individualized, which may influence the occurrence of BCRaL. Neoadjuvant chemotherapy (NAC), a standard treatment for locally advanced breast cancer, has been recognized as a risk factor for BCRaL development (17, 20, 21). Existing prediction models have typically treated chemotherapy as a unified variable without adequately distinguishing between neoadjuvant and adjuvant approaches in risk quantification.

To address this gap, we conducted a retrospective analysis of breast cancer patients receiving both ALND and chemotherapy, with particular focus on differentiating chemotherapy types (neoadjuvant versus adjuvant) as distinct variables. Our study builds upon previous findings by not only validating the independent risk of NAC in a rigorously defined ALND patient cohort but also precisely quantifying its risk relative to adjuvant chemotherapy (AC). By incorporating this distinction into a comprehensive nomogram, we provide an enhanced risk assessment tool that enables more individualized surveillance and prevention strategies for postoperative breast cancer patients, ultimately aiming to reduce BCRaL incidence and improve quality of life.

## Materials and methods

### Patient population

We collected data on primary breast cancer patients who underwent axillary lymph node dissection (ALND) at Shanxi Bethune Hospital, China, from January 2013 to January 2023. A total of 792 patients were initially identified. The inclusion criteria were (1): histopathologically confirmed primary breast cancer (2); clinical stage any T, any N, M0; and (3) chemotherapy treatment. Patients were excluded if they had bilateral breast cancer, stage M1 at initial diagnosis, received fewer than four chemotherapy courses or none at all, underwent chest wall radiotherapy before surgery, had arm lymphedema before surgery, or were lost to follow-up, experienced recurrence, or died during data collection. After screening, the following patients were excluded: 27 with initial stage M1 breast cancer, 5 with bilateral breast cancer, 25 who did not receive chemotherapy, 6 who received only one or two courses of chemotherapy, 62 lost to follow-up, 79 who died, and 53 with recurrent disease. A total of 535 patients were included in the study.

Among these patients, 160 underwent modified radical mastectomy (MRM), and 375 underwent breast-conserving surgery (BCS). All patients received four to eight courses of chemotherapy, primarily consisting of anthracyclines or taxanes, with 150 patients receiving neoadjuvant chemotherapy (NAC). Postoperatively, 335 patients received radiotherapy (RT), of whom 218 (65.1%) underwent supraclavicular radiotherapy (SCRT). Hormone receptor-positive (HR+) patients underwent adjuvant endocrine therapy using selective estrogen receptor modulators (SERMs) or aromatase inhibitors (AIs). Of the 163 human epidermal growth factor receptor 2-positive (HER2+) patients, 132 (81.0%) were treated with trastuzumab, either alone or with pertuzumab.

### BCRaL assessments

Patients were required to return to the hospital for follow-up every 4–6 months after breast cancer surgery. During these follow-ups, in addition to assessing for recurrence, arm lymphedema was also evaluated by trained international lymphedema therapists. We measured the circumference of both upper limbs using a soft tape measure at six sites: the hand, wrist, midpoint between wrist and elbow, elbow joint, midpoint between elbow joint and axilla, and the axilla. The BCRaL was defined as the circumference of the affected arm being 2 cm or greater than the circumference of the contralateral arm at any of the above measurement points.

For patients who did not attend regular follow-up visits, we conducted telephone follow-up visits. We urged patients to return for regular follow-ups and asked if they had any unusual sensations, such as swelling or numbness in the affected limb, or if they had noticed a difference in the circumference of the arm. If an abnormality above was found, we would recall the patient to the hospital for further measurement of the arm circumference. The last follow-up visit was conducted in January 2024.

All enrolled patients underwent standardized BCRaL monitoring with uniform follow-up intervals, assessment protocols, and diagnostic criteria. Only patients with complete follow-up data were included in the analysis. Lymphedema-free survival was defined as the time interval from the date of surgery to the date of BCRaL diagnosis or the last follow-up.

### Statistical analyses

Normally distributed data were expressed as mean ± standard deviation, and an independent sample t-test compared continuous variable groups. For non-normally distributed data, the median (interquartile range, IQR) [M (P25, P75)] was used for description, and group comparisons were performed using the rank-sum test. Categorical data were summarized with counts and percentages, and group comparisons utilized the chi-squared test. When the chi-squared test was not applicable, Fisher’s exact test was used. Missing values were imputed using multiple imputations with the “missRanger” package in R. Subjects were randomly divided into a training set (70%) and a validation set (30%) using the ‘sample’ function in R, with the training set designated for model training and the validation set for model validation.

The prediction model was constructed using the training set by performing univariate Cox regression analysis to identify potential predictors of the outcome event (P < 0.05). The identified variables were then included in a multivariate Cox regression analysis (stepwise, bidirectional) to explore independent predictors (*P* < 0.05). The prediction model nomogram was constructed based on the independent predictors. The proportional hazards assumption for each variable was tested in the final model using Schoenfeld’s residual test, with P > 0.05, indicating that the proportional hazards assumption was satisfied. The constructed nomogram underwent five-fold cross-validation, and calibration curves were drawn to evaluate the model’s calibration. A time-dependent receiver operating characteristic (ROC) curve analysis was conducted to determine the area under the curve (AUC), C-index, and additional metrics for evaluating the model’s discriminative performance. Clinical decision curve analysis (DCA) was performed to assess the model’s clinical utility and measure net benefits across the threshold probability range. Finally, the constructed nomogram model was further validated using the validation set data. Additionally, the nomogram scores for all subjects were calculated based on the model. Using the ‘surv cutpoint’ function from the ‘survminer’ package in R, the optimal cut-off value for the nomogram score was identified in the training set. Subjects were subsequently categorized into high- and low-risk groups based on this cut-off, and Kaplan-Meier curves were generated for log-rank tests to validate the findings.

Analyses were conducted using R version 4.3.2. The “rms” package was used for constructing and drawing the nomogram, the “QHScrnomo” package for drawing calibration curves, the “riskRegression”, “ggprism”, and “ggplot2” packages for drawing time-dependent ROC curves, the “ggDCA” package for clinical decision curve analysis, the “nomogramFormula” package for calculating nomogram scores, and the “survminer” package for survival analysis. All tests were two-sided, with statistical significance set at *P* < 0.05.

## Results

### Patient clinicopathological characteristics

The study included 535 patients, divided into a training cohort of 374 and a validation cohort of 161. At a median follow-up of 38.97 [19.00, 68.33] months, the overall incidence of BCRaL was 20.6%, with similar incidences in both the training (20.6%) and validation (20.5%) cohorts (*P* = 0.981). The median time to lymphedema was 12.40 [7.43, 22.78] months. The patients had a median age of 51 years (IQR: 44-59) and an average BMI of 24.76 ± 3.25. The median (IQR) axillary lymph nodes removed was 21 [16, 27]. **Table 1** outlines the clinicopathological characteristics for both the training and validation sets. The two cohorts showed no significant differences in variables other than educational level (*P* = 0.048) and clinical N stage (*P* = 0.030).

**Table 1:** Clinicopathological characteristics of patients in the training and validation cohorts.

Variables	Total cohort (n = 535)	Training cohort (n = 374)	Validation cohort (n = 161)	*P* value
Lymphedema, No. (%)				0.981
No	425 (79.4)	297 (79.4)	128 (79.5)	
Yes	110 (20.6)	77 (20.6)	33 (20.5)	
Age, Median (IQR)	51 (44, 59)	50 (43, 59)	52 (44, 59)	0.461
BMI, Mean ± SD	24.76 ± 3.25	24.77 ± 3.29	24.75 ± 3.16	0.955
Educational level, No. (%)				0.048
Junior level and below	420 (78.5)	285 (76.2)	135 (83.9)	
Senior level and above	115 (21.5)	89 (23.8)	26 (16.1)	
Menopausal status, No. (%)				0.401
No	274 (51.2)	196 (52.4)	78 (48.4)	
Yes	261 (48.8)	178 (47.6)	83 (51.6)	
Clinical T stage, No. (%)				0.166
T0, T1	200 (37.4)	135 (36.1)	65 (40.4)	
T2	285 (53.3)	209 (55.9)	76 (47.2)	
T3	35 (6.5)	20 (5.3)	15 (9.3)	
T4	15 (2.8)	10 (2.7)	5 (3.1)	
Clinical N stage, No. (%)				0.030
N0	323 (60.4)	238 (63.6)	85 (52.8)	
N1	149 (27.9)	93 (24.9)	56 (34.8)	
N2	45 (8.4)	28 (7.5)	17 (10.6)	
N3	18 (3.4)	15 (4)	3 (1.9)	
HR status, No (%)				0.671
No	107 (20)	73 (19.5)	34 (21.1)	
Yes	428 (80)	301 (80.5)	127 (78.9)	
HER2 status, No. (%)				0.407
No	372 (69.5)	256 (68.4)	116 (72)	
Yes	163 (30.5)	118 (31.6)	45 (28)	
Molecular subtype, No (%)				0.139
HR+/HER2-	323 (60.4)	228 (61)	95 (59)	
HR+/HER2+	105 (19.6)	73 (19.5)	32 (19.9)	
HR-/HER2+	58 (10.8)	45 (12)	13 (8.1)	
HR-/HER2-	49 (9.2)	28 (7.5)	21 (13)	
Breast surgery, No (%)				0.813
BCS	160 (29.9)	113 (30.2)	47 (29.2)	
MRM	375 (70.1)	261 (69.8)	114 (70.8)	
Breast reconstruction, No (%)				0.225
No	488 (91.2)	339 (90.6)	149 (92.5)	
Autologous	29 (5.4)	24 (6.4)	5 (3.1)	
Implant	18 (3.4)	11 (2.9)	7 (4.3)	
Axillary surgery, No (%)				0.287
SLNB+ALND	277 (51.8)	188 (50.3)	89 (55.3)	
ALND	258 (48.2)	186 (49.7)	72 (44.7)	
Axillary lymph nodes removed, Median (IQR)	21 (16, 27)	20 (15, 26)	21 (17, 28)	0.276
Axillary positive lymph nodes, Median (IQR)	2 (1, 4)	2 (1, 4)	2 (1, 4)	0.969
Chemotherapy, No (%)				0.977
AC	385 (72)	269 (71.9)	116 (72)	
NAC	150 (28)	105 (28.1)	45 (28)	
Taxane-based chemotherapy, No (%)				0.633
No	42 (7.9)	28 (7.5)	14 (8.7)	
Yes	493 (92.1)	346 (92.5)	147 (91.3)	
HER2-targeted therapy, No (%)				0.552
No	403 (75.3)	279 (74.6)	124 (77)	
Yes	132 (24.7)	95 (25.4)	37 (23)	
Radiotherapy, No (%)				0.874
No	200 (37.4)	139 (37.2)	61 (37.9)	
Yes	335 (62.6)	235 (62.8)	100 (62.1)	
SCRT, No (%)				0.758
No	317 (59.3)	220 (58.8)	97 (60.2)	
Yes	218 (40.7)	154 (41.2)	64 (39.8)	
Endocrine therapy, No (%)				0.862
No	107 (20)	73 (19.5)	34 (21.1)	
SERM	80 (15)	55 (14.7)	25 (15.5)	
AI	348 (65)	246 (65.8)	102 (63.4)	

The clinical stage is the stage before treatment.

BMI, body mass index; T; tumor; N; node; HR, hormone receptor; HER2, human epidermal growth factor receptor 2, HR+/HER2, HR-positive and HER2-negative, BCS breast-conserving surgery, MRM modified radical mastectomy; SLNB, sentinel lymph node biopsy; ALND, axillary lymph node dissection; AC, adjuvant chemotherapy; NAC, neoadjuvant chemotherapy; SCRT, supraclavicular radiotherapy; SERM, selective estrogen receptor modulator; AI, aromatase inhibitor; IQR, interquartile range.

### Univariate and multivariate analyses

**Table 2** presents the univariate and multivariate analyses conducted to identify risk factors in the training cohort. Univariate analysis identified associations between BCRaL risk and BMI, clinical N stage, the number of axillary lymph nodes removed, the number of positive axillary lymph nodes, chemotherapy type, HER2-targeted therapy, and SCRT. Multivariate analysis further confirmed the independent risk factors for BCRaL. The BCRaL risk significantly increased with higher BMI (Hazard ratio (HR) = 1.121, 95% confidence interval (CI): 1.046-1.192 per 1 kg/m2 increase; *P* < 0.001), more positive axillary lymph nodes (HR = 1.040, 95% CI: 1.009-1.072; *P* = 0.011), and receipt of NAC (HR = 2.024, 95% CI: 1.248-3.283; *P* = 0.004), HER2-targeted therapy (HR = 1.689, 95% CI: 1.026-2.779; *P* = 0.039), or SCRT (HR = 1.666, 95% CI: 1.033-2.687; *P* = 0.036).

**Table 2:** Univariate and multivariate analyses of risk factors in the training cohort.

Variables	Univariate analysis		Multivariable analysis	
	HR (95% CI)	*P* value	HR (95% CI)	*P* value
Age, per 1-year increase	1.012 (0.991, 1.034)	0.270		
BMI, per 1 increase	1.117 (1.046, 1.192)	0.001	1.121 (1.053, 1.194)	<0.001
Educational level				
Junior level and below	reference			
Senior level and above	1.233 (0.752, 2.022)	0.406		
Menopausal status				
No	reference			
Yes	1.256 (0.803, 1.964)	0.318		
Clinical T stage				
T0, T1	reference			
T2	1.465 (0.883, 2.431)	0.140		
T3	2.071 (0.840, 5.110)	0.114		
T4	1.478 (0.347, 6.292)	0.597		
Clinical N stage				
N0	reference			
N1	1.524 (0.906, 2.565)	0.113		
N2	2.066 (1.002, 4.263)	0.050		
N3	2.979 (1.261, 7.035)	0.013		
HR status				
No	reference			
Yes	0.682 (0.410, 1.135)	0.141		
HER2 status				
No	reference			
Yes	1.478 (0.934, 2.338)	0.095		
Molecular subtype				
HR+/HER2-	reference			
HR+/HER2+	1.561 (0.892, 2.732)	0.119		
HR-/HER2+	1.630 (0.854, 3.114)	0.139		
HR-/HER2-	1.689 (0.789, 3.614)	0.177		
Breast surgery				
BCS	reference			
MRM	0.779 (0.487, 1.244)	0.295		
Breast reconstruction				
No	reference			
Autologous	1.348 (0.585, 3.106)	0.483		
Implant	0.518 (0.072, 3.739)	0.514		
Axillary surgery				
SLNB+ALND	reference			
ALND	1.311 (0.834, 2.059)	0.240		
Axillary lymph nodes removed, per 1-lymph node increase	1.027 (1.004, 1.051)	0.022		
Axillary positive lymph nodes, per 1-lymph node increase	1.049 (1.019, 1.079)	0.001	1.040 (1.009, 1.072)	0.011
Chemotherapy				
AC	reference		reference	
NAC	2.344 (1.492, 3.684)	<0.001	2.024 (1.248, 3.283)	0.004
Taxane-based chemotherapy				
No	reference			
Yes	2.203 (0.694, 6.994)	0.180		
HER2-targeted therapy				
No	reference		reference	
Yes	1.795 (1.127, 2.857)	0.014	1.689 (1.026, 2.779)	0.039
Radiotherapy				
No	reference			
Yes	1.502 (0.891, 2.531)	0.127		
SCRT				
No	reference		reference	
Yes	1.943 (1.230, 3.069)	0.004	1.666 (1.033, 2.687)	0.036
Endocrine therapy				
No	reference			
SERM	0.444 (0.195, 1.009)	0.053		
AI	0.748 (0.444, 1.260)	0.275		

The clinical stage is the stage before treatment.

BMI, body mass index; T; tumor; N; node; HR, hormone receptor; HER2, human epidermal growth factor receptor 2, HR+/HER2- HR-positive and HER2-negative; BCS, breast-conserving surgery; MRM, modified radical mastectomy; SLNB, sentinel lymph node biopsy; ALND, axillary lymph node dissection; AC, adjuvant chemotherapy; NAC, neoadjuvant chemotherapy; SCRT, supraclavicular radiotherapy; SERM, selective estrogen receptor modulator; AI, aromatase inhibitor; HR, Hazard ratio.

### Construction and validation of the BCRaL nomogram

**Figure 1:**
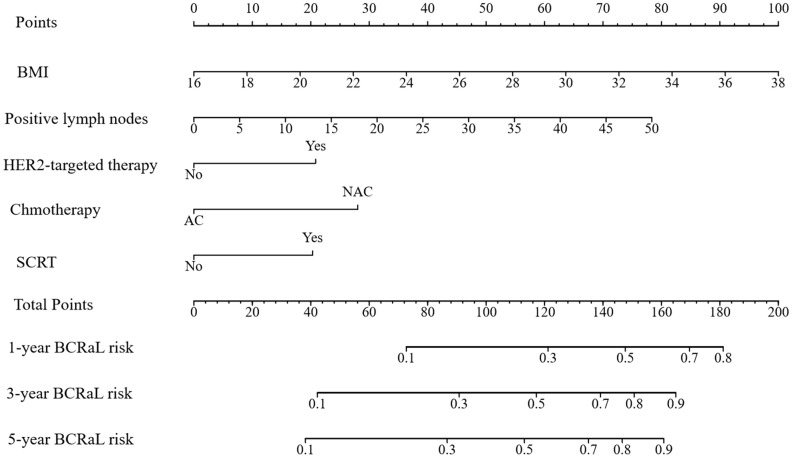
Nomogram for predicting BCRaL risk. This nomogram predicts 1-year, 3-year, and 5-year BCRaL risk based on several clinical variables, including Body Mass Index (BMI), the number of positive lymph nodes, chemotherapy type (AC or NAC), HER2-targeted therapy, and SCRT. Each variable is assigned points, with total points indicating the estimated BCRaL risk.

**Figure 2:**
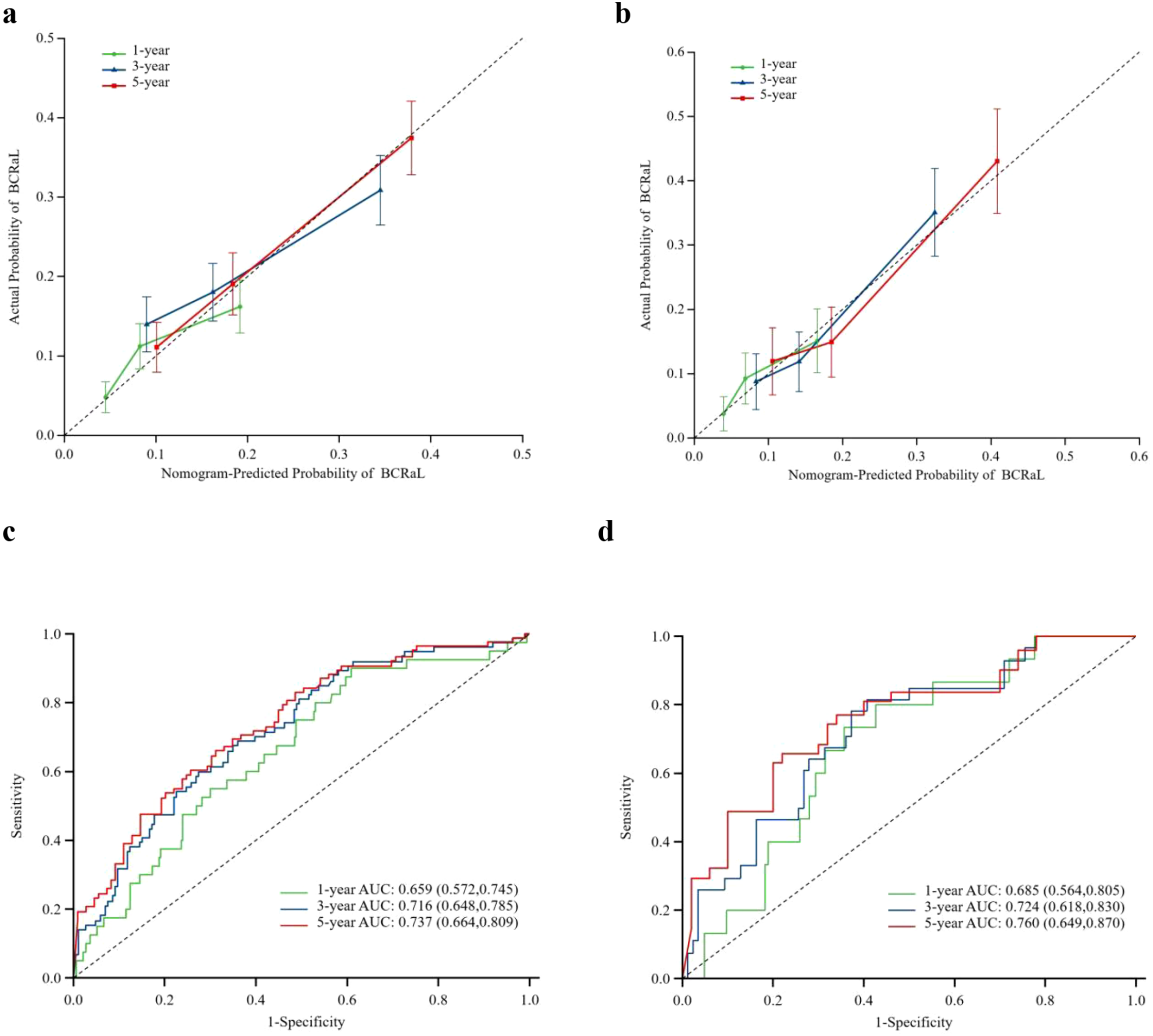
Calibration curves for predicting BCRaL risk in the training set **(a)** and validation set **(b)** The curves compare nomogram-predicted and actual probabilities of BCRaL at 1, 3, and 5 years. The X-axis labels represent the patient’s BCRaL risk as calculated by the model, typically ranging from 0% to 100% (or 0 to 1.0). The Y-axis labels represent patient cohorts with corresponding predicted probabilities, indicating the actual incidence rate of BCRaL. The diagonal line is a 45-degree diagonal line originating from the origin. If the model’s predictions are perfectly accurate, all data points should lie on this line. The calibration curve is an actual curve plotted based on the data. This curve shows the corresponding actual occurrence rates at different predicted probability levels. Closer alignment to the diagonal indicates better model accuracy. ROC Curves for Training Set **(c)** and Validation Set **(d)** This figure summarizes the model’s performance using ROC curves for both the training and validation sets.

This study integrated the independent risk factors for BCRaL in the training cohort and constructed a nomogram prediction model for BCRaL risk (**Figure 1**). Each variable received a score based on the points scale. The total score was calculated by summing the scores of each independent variable. The total score predicts the probability of BCRaL at 1, 3, and 5 years in the nomogram. The nomogram’s C-index in the training cohort was 0.692 (95% CI: 0.633-0.751). **Figure 2a** shows that the nomogram’s predicted BCRaL risks at 1, 3, and 5 years align closely with the actual observed risks in the training cohort, demonstrating good calibration. **Figure 2b** confirms the finding in the validation cohort, with the nomogram achieving a C-index of 0.719 (95% CI: 0.640-0.797), indicating strong concordance between observed outcomes and predictions.

**Figures 2c, d** display the ROC curves for the training and validation cohorts, respectively. In the training cohort, the AUC values were 0.659 (95% CI: 0.572-0.745) for 1 year, 0.716 (95% CI: 0.648-0.785) for 3 years, and 0.737 (95% CI: 0.664-0.809) for 5 years. In the validation cohort, the AUC values were 0.685 (95% CI: 0.564-0.805) for 1 year, 0.724 (95% CI: 0.618-0.830) for 3 years, and 0.760 (95% CI: 0.649-0.870) for 5 years. The model demonstrated strong discriminative ability in both cohorts. **Appendix Figure 1** presents the time-dependent ROC curves for the combined training and validation cohorts. The model’s high concordance index at various follow-up times validates its prediction accuracy.

### Clinical application of the BCRaL nomogram

**Figure 3:**
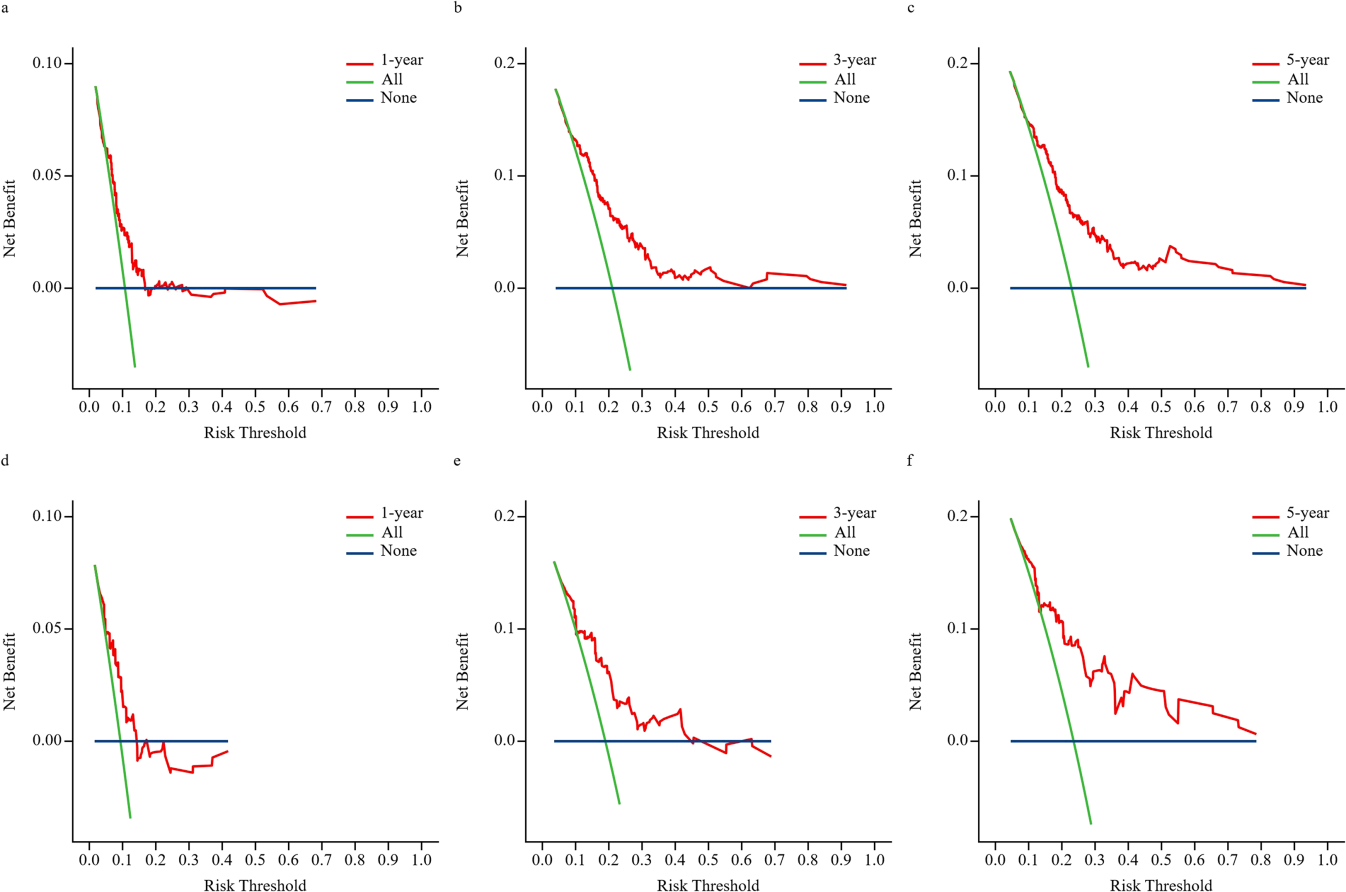
DCA for 1-year, 3-year, and 5-year predictions. This figure presents DCA to evaluate the clinical utility of the nomogram model at different risk thresholds for 1-year, 3-year, and 5-year outcomes in the training set (**a–c**) and validation set (**d–f**). The “All” represents applying the intervention strategy to all patients; as the risk threshold increases, net benefit diminishes due to the overtreatment of low-risk individuals. “None” represents the strategy of not implementing any intervention, consistently yielding zero net benefit. The “Nomogram (red curve)” represents the net benefit of using this prediction chart to guide decisions—that is, intervening only in patients predicted to be high-risk.

The DCA curves in **Figure 3** display the clinical utility and net benefit of the nomogram at predicting the BCRaL risk at different time points (**Figures 3a–c** for the training cohort, **Figures 3d–f** for the validation cohort). The results indicated that using the nomogram for decision-making yields a higher net benefit compared to two extreme strategies: intervening in all patients (All) and intervening in no patients (None).

In the training cohort, the optimal nomogram score cut-off was determined to be 82.69 (**Appendix Figure 2**). This criterion classified patients into high- and low-risk groups for BCRaL. **Appendix Figure 3** displays the survival curves derived from the nomogram scores for the training, validation, and entire cohorts, respectively. The HR values were statistically significant across all cohorts: training cohort (HR = 3.46, 95% CI: 2.21-5.41, *P* < 0.001), validation cohort (HR = 2.78, 95% CI: 1.40-5.53, *P* = 0.002), and total cohort (HR = 3.24, 95% CI: 2.23-4.71, *P* < 0.001). Patients in the high-risk group are significantly more likely to develop BCRaL than those in the low-risk group.

## Discussion

This retrospective cohort study, with a median follow-up of 39 months, established a 20.6% incidence of BCRaL following ALND and chemotherapy. We developed and internally validated a BCRaL risk prediction model that specifically differentiates between NAC and AC as distinct variables. In addition to NAC, the model identifies elevated BMI, an increased number of positive lymph nodes, HER2-targeted therapy, and SCRT as independent predictors. By integrating these factors into a comprehensive nomogram, this study provides a validated tool that incorporates NAC to enable individualized risk assessment and supports targeted prevention strategies for patients undergoing ALND and chemotherapy.

Our investigation demonstrated that NAC significantly elevated BCRaL risk, showing a two-fold increase compared to AC. This finding aligns with existing literature, including a prospective study by Montagna et al. demonstrating NAC to be independently associated with BCRaL risk versus upfront surgery (OR = 2.10, 95% CI: 1.16-3.95) (22). Another investigation reported an even more pronounced 3.76-fold risk elevation (95% CI: 2.29-6.20) among ALND patients receiving NAC (23). Given the established role of NAC in the preoperative management of locally advanced breast cancer and selected triple-negative or HER2-positive subtypes (24, 25), a comprehensive understanding of its associated risks is clinically imperative. The elevated risk may be attributable to multimodal treatment sequencing. As proposed by Kim et al. (26), patients undergoing NAC typically receive radiotherapy shortly following ALND, potentially inducing secondary lymphatic injury before adequate collateral circulation develops, thereby compromising recovery. In contrast, Specht et al. observed a non-significant trend toward risk reduction with NAC (HR = 0.74, 95% CI: 0.37-1.48), hypothesizing that tumor downstaging might mitigate lymphatic damage (27).

The analysis further identified HER2-targeted therapy as a significant predictor, with patients receiving this treatment exhibiting a 1.689-fold increased BCRaL risk. The biological mechanism linking HER2-targeted therapy to BCRaL risk remains unclear. One plausible hypothesis involves vascular endothelial growth factor-C (VEGF-C), a critical mediator of lymphangiogenesis (28, 29). HER2-overexpressing breast cancers demonstrate upregulated VEGF-C expression (30), and anti-HER2 agents have been shown to suppress VEGF-C levels (29, 31). Thus, targeted therapy might inadvertently inhibit lymphatic repair processes. However, it is also critical to consider that HER2-positive disease is characterized by more aggressive features and higher nodal burden. Therefore, while our model adjusted for nodal status, the observed association may partially reflect the underlying tumor biology rather than a direct treatment effect.

A strong association emerged between elevated BMI and BCRaL development, with each unit increase in BMI corresponding to a hazard ratio of 1.121. This study confirmed a significant association between elevated BMI and increased BCRaL risk (HR = 1.121), consistent with established literature (14, 15, 19, 32, 33). The BMI definitions vary across studies, with Jiang et al. establishing BMI>25 kg/m² as a risk threshold (OR = 1.73, 95%CI:1.30-2.31) (9), and Armer et al. identified continuous BMI increase as an independent risk factor (HR = 1.03, 95%CI:1.01-1.06) (20). Our findings demonstrate progressive risk elevation with rising BMI. Additional studies validate the prognostic utility of continuous BMI for predicting lymphedema severity (34). The underlying mechanisms likely involve dual pathways: obesity-induced inflammatory factors impair lymphatic structure and function, increasing leakage while decreasing pumping capacity (35–38); simultaneously, excess adipose tissue may mechanically compress the lymphatic system, impeding lymphatic fluid return (36, 39, 40). These pathophysiological changes collectively contribute to elevated lymphedema risk in high-BMI patients.

The study also revealed a dose-response relationship between nodal burden and lymphedema risk, demonstrating a 4% risk increase per additional positive lymph node. Similar findings have been reported in several previous studies (16, 41–44). Penn et al. discovered that an increase of one positive lymph node increased the BCRaL risk by 8% (43). Additionally, a meta-analysis showed a moderate level of evidence supporting the presence of metastatic lymph nodes as a risk factor for BCRaL occurrence (14). Positive lymph nodes may experience an increase in the BCRaL risk due to lymphatic blockage caused by tumoral infiltration (45).

Notably, SCRT was specifically associated with heightened BCRaL risk, whereas RT overall showed no significant association. The extensive axillary dissection in our cohort (median 20 nodes removed) may have obscured any additional risk from general RT, as suggested by Naoum et al. (46). However, we found that SCRT was associated with a nearly 1.7-fold increased BCRaL risk compared to the non-SCRT patient population (HR = 1.666, 95% CI: 1.033-2.687). This is consistent with Jung’s (HR = 1.93, 95% CI: 1.20-3.10) and Kim’s (HR = 2.74, 95% CI: 1.80-4.17) findings that breast RT with SCRT resulted in a greater risk of lymphedema development (47, 48). Anatomically, the supraclavicular region contains dense lymphatic networks, and SCRT likely impairs drainage capacity through tissue fibrosis and direct vessel damage (49), explaining its distinct risk profile compared to RT alone.

We successfully developed and validated a comprehensive nomogram incorporating five independent predictors: elevated body mass index (BMI), increased number of positive axillary lymph nodes, NAC, HER2-targeted therapy, and SCRT. The model demonstrated good discrimination with C-index values of 0.692 (training) and 0.719 (validation), along with consistent time-dependent AUC values (0.659-0.760) across 1-, 3-, and 5-year predictions. Calibration curves confirmed accuracy, while decision curve analysis established clinical utility superior to universal intervention strategies. With an optimal cutoff score of 82.69, the nomogram effectively stratifies patients into distinct risk categories, providing clinicians with a practical tool for individualized risk assessment and targeted preventive care in breast cancer management. A comparison of our model’s performance with recently published nomograms provides important context. The discriminative ability of our model (AUC 0.760 at 5 years) is lower than the exceptional performance (AUC 0.944) reported by Luo et al. in a large Chinese cohort (13). This discrepancy is likely attributable to fundamental differences in predictor selection and modeling approach. The model by Luo et al. incorporated a wider array of strong predictors, including comorbidities and postoperative complications, and aimed to predict risk within a fixed 12-month period using logistic regression. In contrast, our Cox model focused on treatment-specific factors and provides dynamic risk stratification over a 5-year horizon, a more complex predictive task. Our performance is, however, comparable to the validation AUC of 0.724 reported by Jiang et al. (9). Therefore, the clinical value of our nomogram is its specific focus on quantifying the impact of modern treatment modalities (e.g., NAC, targeted therapy) on long-term lymphedema risk, offering a complementary tool for personalized surveillance planning in high-risk patients undergoing contemporary multi-modal therapy.

Based on the established nomogram cut-off score of 82.69, we propose distinct clinical management pathways: Low-risk patients (score <82.69) receive standardized lymphedema education. High-risk patients (score ≥82.69) are recommended a comprehensive prevention strategy including intensified 3–4 month surveillance for 2–3 years, preventive compression sleeve use during strenuous activities, and weight management support for those with elevated BMI.

The developed prediction model offers several distinct advantages for clinical practice. First, it precisely targets high-risk patients undergoing ALND combined with chemotherapy, ensuring service to those most needing individualized management. Second, it innovatively integrates modern comprehensive treatment elements, including NAC and HER2-targeted therapy. Third, it provides multi-timepoint dynamic risk assessment at 1, 3, and 5 years, overcoming the limitations of traditional static prediction and supporting long-term management strategy development. Fourth, clinical decision curve analysis confirms the model’s significant net benefit and superior clinical utility compared to extreme intervention strategies. While demonstrating clinical utility, this study has several limitations that should be considered when interpreting the results. First, the assessment of BCRaL was based on circumferential measurements, which may introduce measurement bias. Although all measurements were performed by trained therapists using a standardized protocol, potential inter-observer variability remains a concern. Second, due to the retrospective nature of this study, several potentially important predictors were not available for analysis, including the precise level of ALND, operative time, surgical site infection, post-operative complications, and specific comorbidities such as hypertension; other potential risk factors, such as genetic background, lifestyle psychological factors, and financial factors (14, 50, 51), were not included in the model. The omission of these variables may have influenced the observed associations. Third, while we adjusted for key clinicopathological factors, including the number of positive lymph nodes, residual confounding may persist. Specifically, HER2-targeted therapy and SCRT are often indicators of more advanced disease, and despite our multivariate adjustments, it remains challenging to fully disentangle their independent effects from the underlying tumor biology and overall treatment intensity. Fourth, although the model showed strong performance during 1, 3, and 5-year follow-up periods, breast cancer patients may need extended monitoring to evaluate late complications or chronic lymphedema development. Thus, future studies should extend the follow-up period to verify the model’s predictive ability over a longer time frame. Fifth, as this was a single-center study conducted in a Chinese population, the generalizability of our nomogram to other ethnic groups and healthcare settings requires validation. Future external validation in multi-center and international cohorts is essential to ensure the model’s broad applicability and stability. Sixth, with the ongoing evolution of axillary management toward less invasive approaches, the applicability of our model may be most relevant for patients who still require comprehensive ALND due to advanced nodal disease. Finally, the study lacked a pre-specified sample size calculation, a common limitation in retrospective modeling, highlighting the need for confirmation in future, explicitly powered prospective studies.

## Conclusion

This study successfully developed and internally validated a predictive nomogram for BCRaL risk specifically targeting patients undergoing ALND and chemotherapy. The model innovatively integrates modern treatment modalities (NAC, HER2-targeted therapy, SCRT) and traditional risk factors (BMI, number of positive lymph nodes), enabling precise identification of high-risk patients. Validation results demonstrated robust predictive accuracy and clinical utility, with consistent performance maintained across 1-, 3-, and 5-year follow-up periods. It is important to note that this model was not externally validated, making its implementation a critical future research step. The proposed tool should be used only as a reference for identifying high-risk patients. Its clinical utility in guiding precision prevention strategies remains exploratory and requires validation through future multicenter studies.

## Data Availability

The original contributions presented in the study are included in the article/supplementary material. Further inquiries can be directed to the corresponding authors.
