# Supplementary material for: Nomogram for predicting risk of arm lymphedema following axillary lymph node dissection in breast cancer patients
Source: Front Oncol. 2025 Nov 21;15:1667939. doi: 10.3389/fonc.2025.1667939 (PMC12678132; doi:10.3389/fonc.2025.1667939)
Supplement: Supplementary file 1 [file DataSheet1.docx]

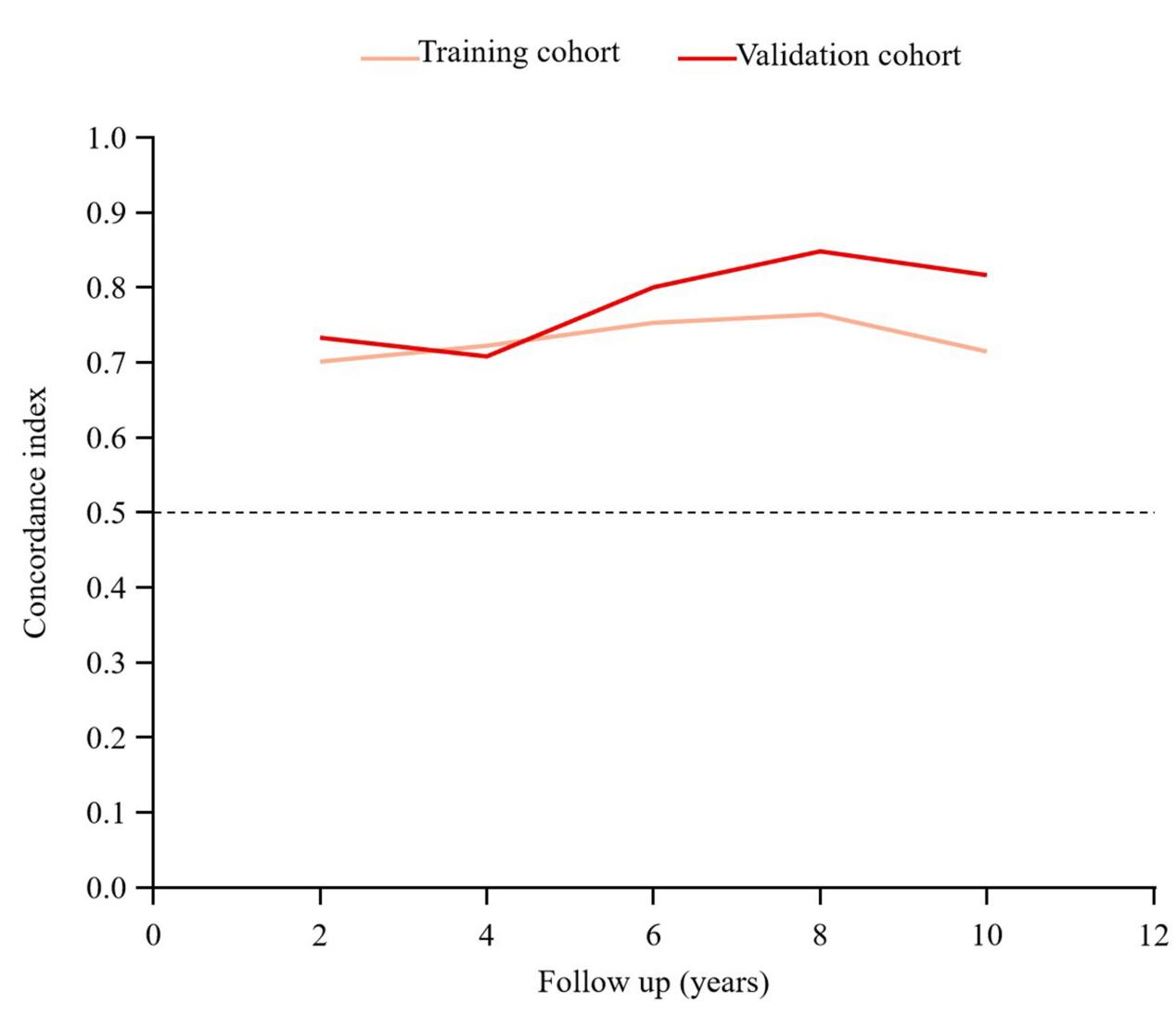


**Appendix Fig 1. Time-Dependent ROC for Training and Validation Sets.** This figure depicts the dynamic performance of the model across different follow-up times, showcasing the time-dependent ROC curves for both the training and validation sets.
