# Supplementary material for: Nomogram for predicting risk of arm lymphedema following axillary lymph node dissection in breast cancer patients
Source: Front Oncol. 2025 Nov 21;15:1667939. doi: 10.3389/fonc.2025.1667939 (PMC12678132; doi:10.3389/fonc.2025.1667939)
Supplement: Supplementary file 2 [file DataSheet2.docx]

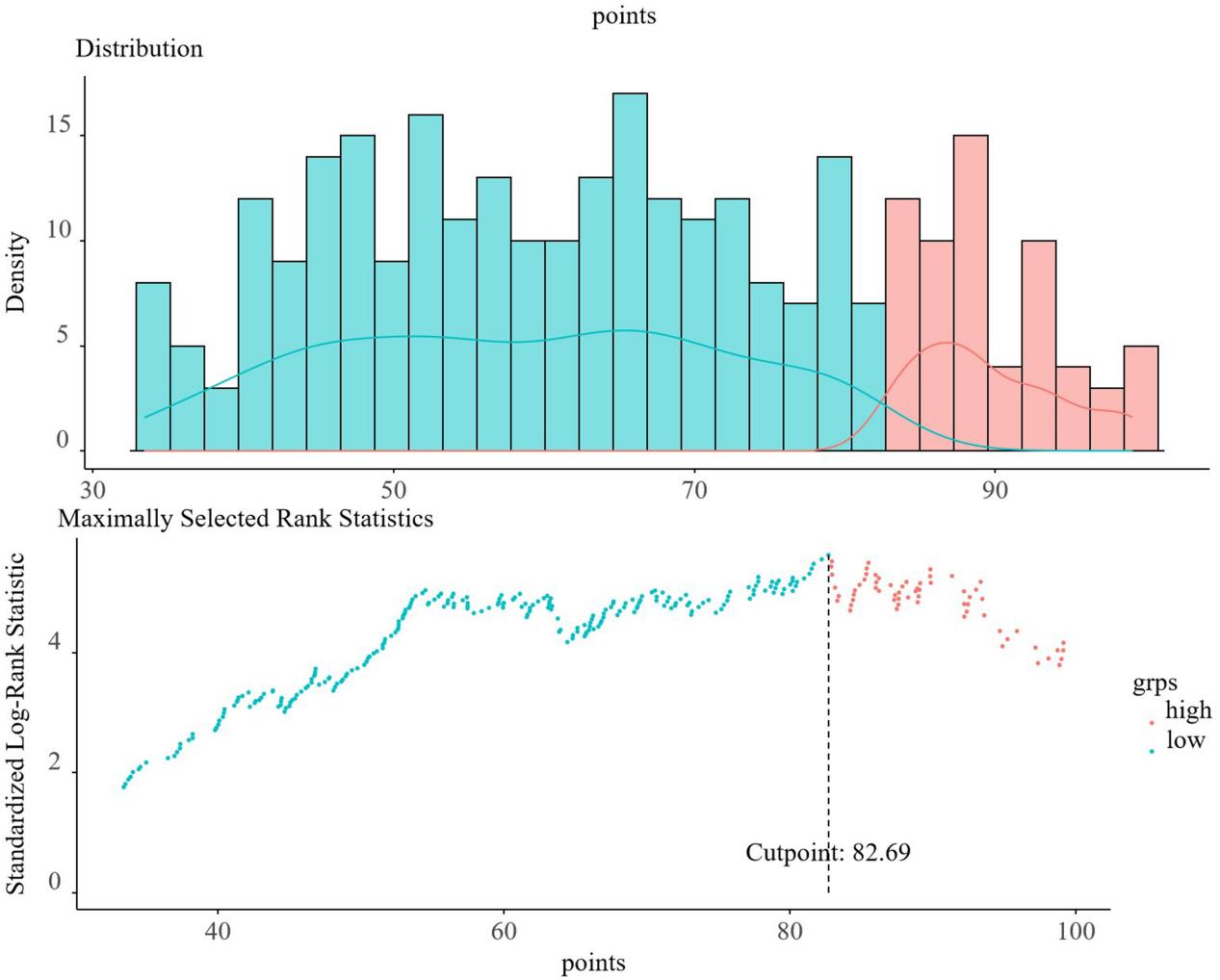


**Appendix Fig 2. Distribution of Nomogram Scores and Risk Group Cutpoint.** This figure illustrates the distribution of nomogram scores and the cutpoint used for dividing patients into high- and low-risk groups based on maximally selected rank statistics. A vertical line marks the cutpoint at 82.69, which separates the patients into high- and low-risk groups.
