# Supplementary material for: Nomogram for predicting risk of arm lymphedema following axillary lymph node dissection in breast cancer patients
Source: Front Oncol. 2025 Nov 21;15:1667939. doi: 10.3389/fonc.2025.1667939 (PMC12678132; doi:10.3389/fonc.2025.1667939)
Supplement: Supplementary file 3 [file DataSheet3.docx]

**a**

**
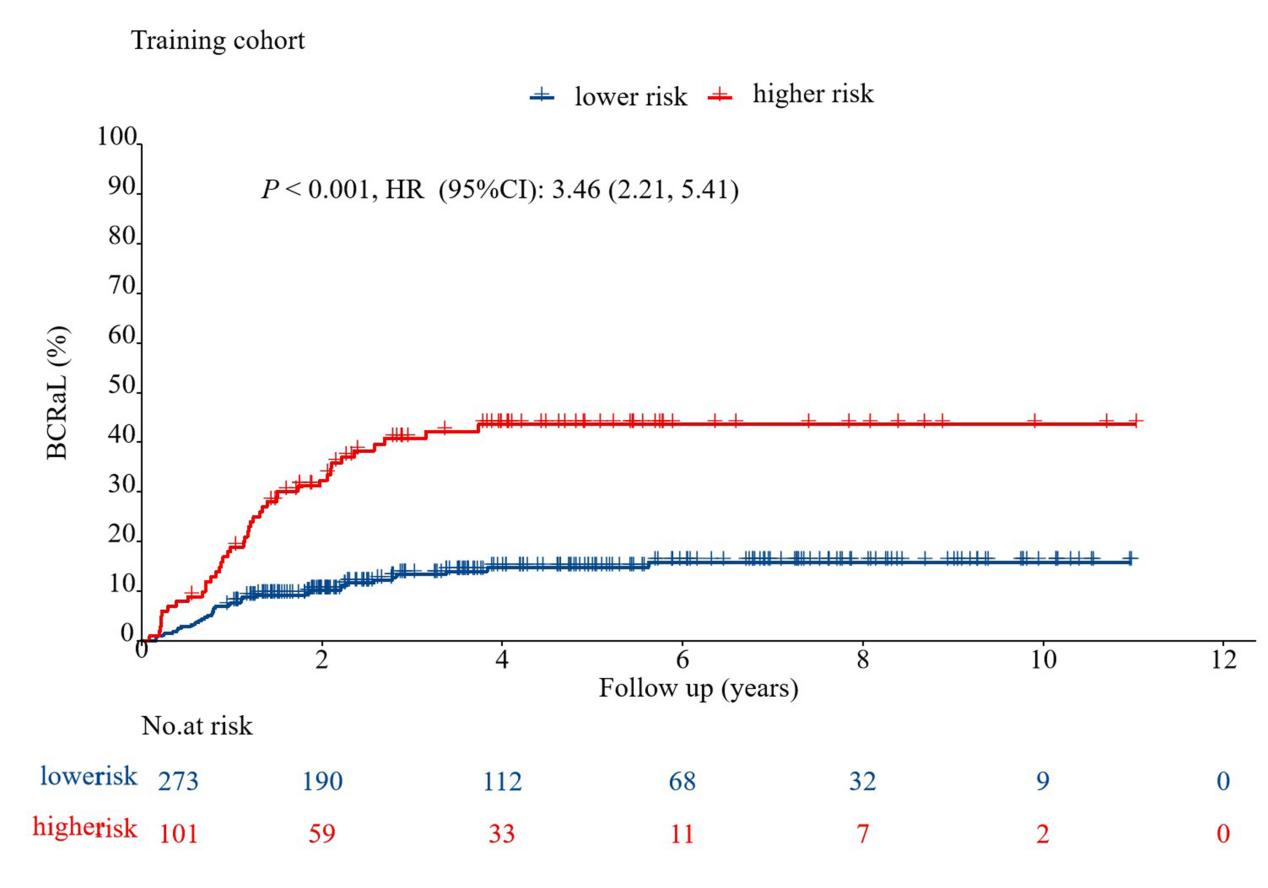
**

**b**

**
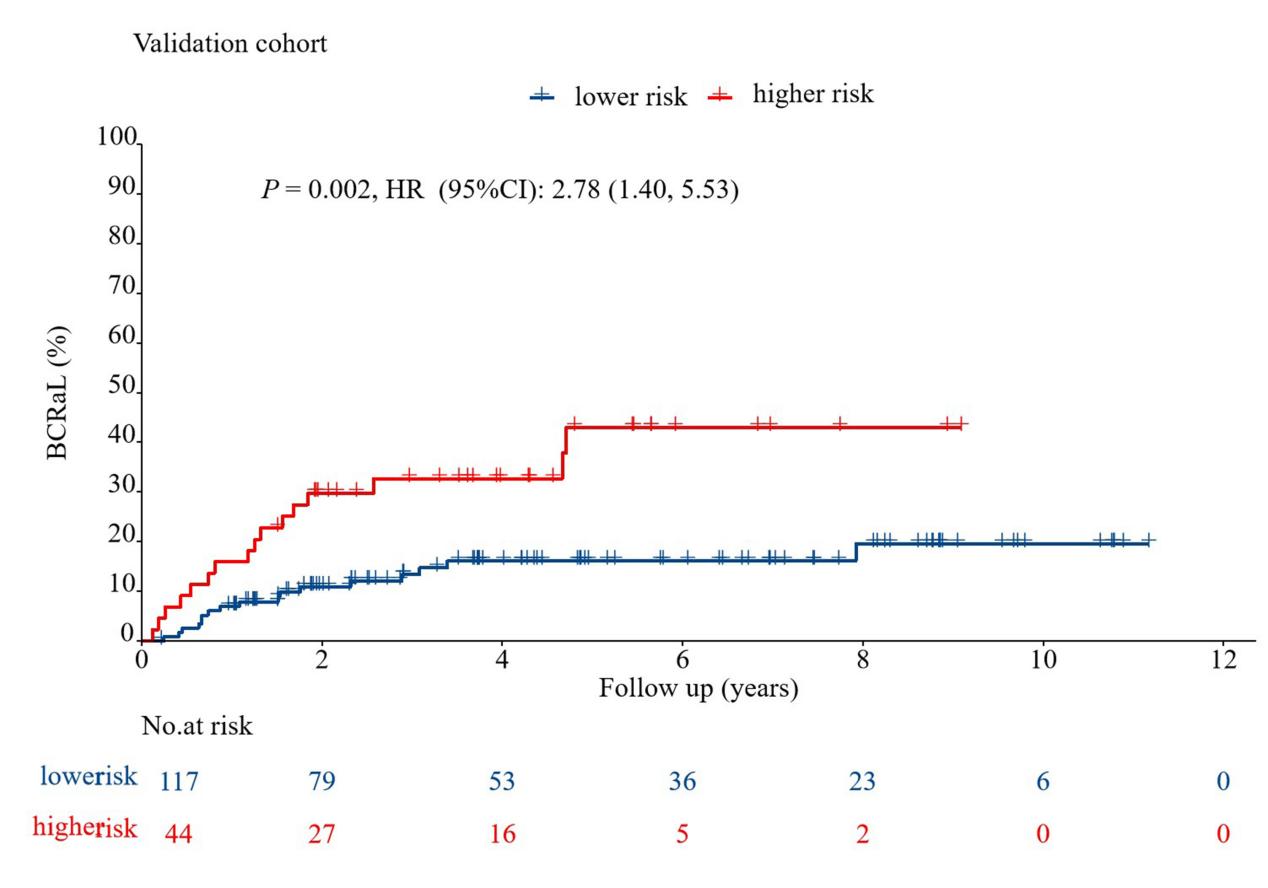
**

**c**

**
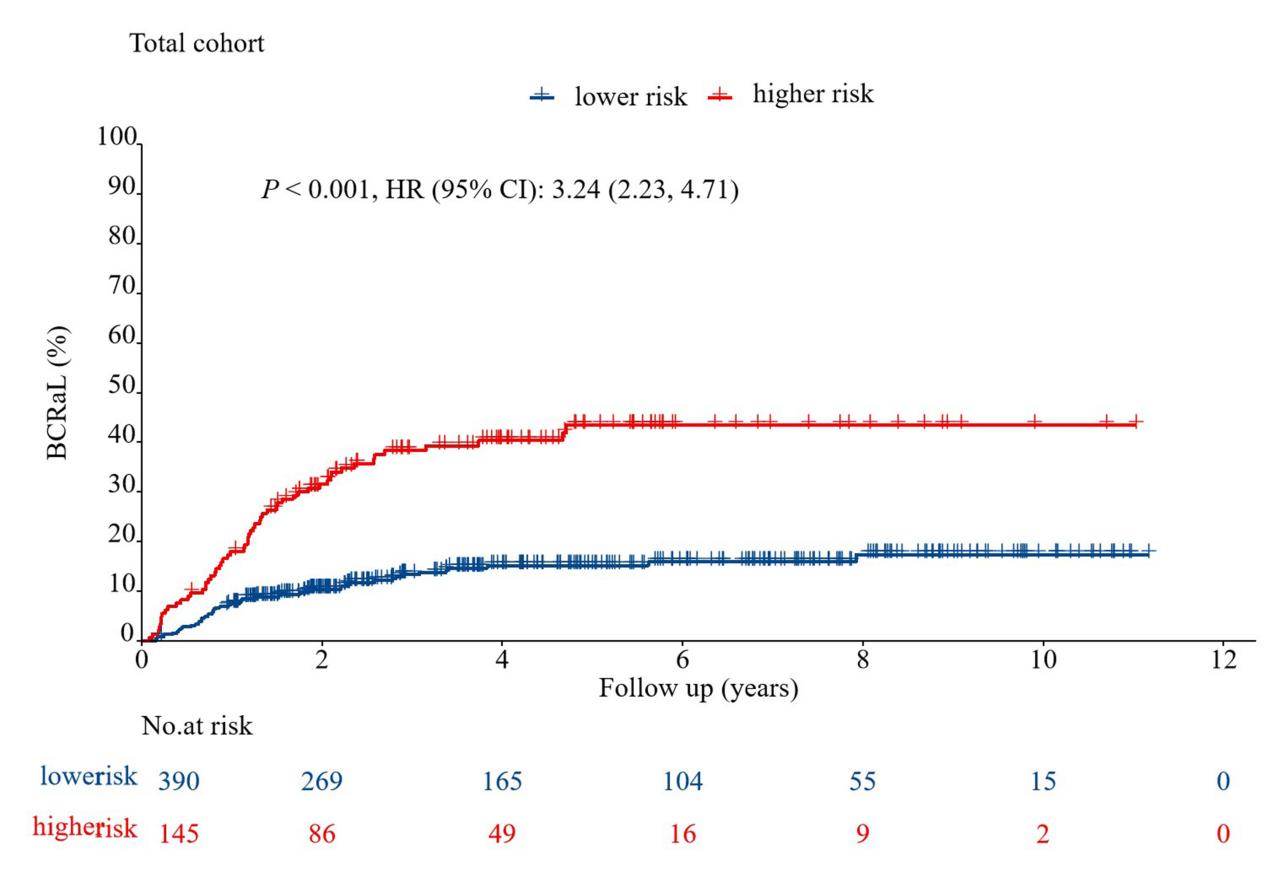
**

**Appendix Fig 3. Kaplan-Meier Survival Curves Based on Nomogram Scores.** This figure shows Kaplan-Meier survival curves for the training (**a**), validation (**b**), and total cohorts (**c**), dividing patients into high- and low-risk groups.
